# Coordinated Molecular Cross-Talk between *Staphylococcus aureus*, Endothelial Cells and Platelets in Bloodstream Infection

**DOI:** 10.3390/pathogens4040869

**Published:** 2015-12-05

**Authors:** Carolina D. Garciarena, Tony M. McHale, Rebecca L. Watkin, Steven W. Kerrigan

**Affiliations:** Cardiovascular Infection Research Group, School of Pharmacy & Molecular and Cellular Therapeutics, Royal College of Surgeons in Ireland, 123 St. Stephen’s Green, Dublin 2, Ireland; E-Mails: carolinagarciarena@rcsi.ie (C.D.G.); tonymchale@rcsi.ie (T.M.M.); rebeccawatkin@rcsi.ie (R.L.W.)

**Keywords:** *Staphylococcus aureus*, platelets, endothelial cells, sepsis, infective endocarditis

## Abstract

*Staphylococcus aureus* is an opportunistic pathogen often carried asymptomatically on the human body. Upon entry to the otherwise sterile environment of the cardiovascular system, *S. aureus* can lead to serious complications resulting in organ failure and death. The success of *S. aureus* as a pathogen in the bloodstream is due to its ability to express a wide array of cell wall proteins on its surface that recognise host receptors, extracellular matrix proteins and plasma proteins. Endothelial cells and platelets are important cells in the cardiovascular system and are a major target of bloodstream infection. Endothelial cells form the inner lining of a blood vessel and provide an antithrombotic barrier between the vessel wall and blood. Platelets on the other hand travel throughout the cardiovascular system and respond by aggregating around the site of injury and initiating clot formation. Activation of either of these cells leads to functional dysregulation in the cardiovascular system. In this review, we will illustrate how *S. aureus* establish intimate interactions with both endothelial cells and platelets leading to cardiovascular dysregulation.

## 1. Bloodstream Infection

*Staphylococcus aureus* is an opportunistic pathogen known for causing a variety of self-limiting and life threatening conditions in the human [1]. These conditions range from mild localised skin infections to severe and potentially fatal invasive infections, including bloodstream infection [2]. The danger of *S. aureus* invasive disease is compounded by the widespread global occurrence of multiple antibiotic resistant strains, such as methicillin and vancomycin resistant *S. aureus*, (MRSA and VRSA, respectively) a phenomenon directly attributed to prolonged use of antibiotics. Upon entry to the bloodstream, *S. aureus* is capable of causing a localised infection such as Infective Endocarditis (IE) or a generalised infection such as sepsis.

### 1.1. Localised Bloodstream Infection: Infective Endocarditis

IE is a life threatening disease caused by bacterial binding to endothelial surfaces of the heart. Although many species of bacteria have been reported to cause IE the majority of cases are attributable to *S. aureus* IE is characterised by the formation of a septic thrombus or vegetative growth on the surface of the cardiac valve [3,4,5,6]. It typically develops in individuals with a pre-existing cardiac defect that leads to a hemodynamic disturbance or in patients with a prosthetic valve (PV) [7,8]. Areas affected by hemodynamic disturbances often lead to turbulent blood flow resulting in damage to endothelial cells and exposure of the extracellular matrix proteins. Deficient endothelialisation of PV generates a similar outcome and increases the yearly incidence of IE by one thousand times [9,10,11,12]. As part of the natural response to damaged endothelium, platelets and fibrin attach to exposed extracellular matrix proteins in order to initiate repair [13]. However, transient bacteria in the bloodstream or indeed bacteria from a distal source can adhere to the sterile platelet fibrin nidus causing the formation of a thrombogenic surface allowing recruitment of a secondary layer of platelets. This growing thrombus encases the bacteria, protecting them from circulating white blood cells and antibiotic treatment [14,15]. Thrombus formation may lead to aortic valve leaflet perforation that can manifest itself as acute congestive heart failure [16].

Treatment for IE is frequently unsuccessful as the infected nidus harbouring sessile thrombus-protected *S. aureus* is impermeable to antibiotics and often requires combined aggressive multi-antibiotic therapy coupled with surgery to remove the vegetation and/or replace the infected valve [17]. Therapy is deemed successful when all traces of bacteria are eliminated from the bloodstream.

### 1.2. Generalised Bloodstream Infection: Sepsis

Sepsis is a potentially life threatening infection of the cardiovascular system characterised as acute organ dysfunction particularly affecting the respiratory and cardiovascular systems [18]. Sepsis is triggered by a variety of microorganisms, although bacteria are the most common cause. One of the most frequently isolated bacteria is *S. aureus* [19]. Sepsis initially occurs when *S. aureus* gains access to the bloodstream, allowing it to bind to the endothelium. This leads to an impairment of vascular function due to a cytokine storm from immune and endothelial cells. This host response results in vasodilation, increased vascular permeability, cardiac depression and impairment of the coagulation cascade [20]. The loss of barrier function, caused by increased vascular permeability contributes to multiple organ failure as leakage of parenchymal and interstitial fluid from the blood vessels increases the distance for oxygen to reach the organs to allow normal function. Additionally, widespread activation of the coagulation cascade resulting in occlusion of micro vessels by circulating thrombi [21] compromises blood flow and contributes to multiple organ damage in the septic patients. *S. aureus* deposition on damaged or dysfunctional vascular endothelial cells, as in IE, serves as a thrombogenic surface for the recruitment of platelets. This leads to platelet activation and amplification of a growing thrombus resulting in further vascular occlusion.

The principles of the initial management bundle for sepsis patients are to provide cardiorespiratory resuscitation and to mitigate the immediate threat of uncontrolled infection [22,23]. Resuscitation requires administration of intravenous fluids and vasopressors with mechanical ventilation as necessary. Aggressive intravenous combination antimicrobial therapy (several classes of broad spectrum antibiotics covering all likely causative pathogens) for long periods of time is the only available treatment [24]. However with the rapid global emergence of multiple antibiotic resistant strains of bacteria, treatment is becoming increasingly difficult and less successful.

Regardless of infection type (localised or generalised), a common feature of *S. aureus* infection in the bloodstream is the interaction of the bacteria with vascular endothelial cells and/or platelets. Dysregulation of either or both of these host cells contribute significantly to the clinical outcome. The following sections of this review will therefore focus on the known interactions between *S. aureus*, platelets and vascular endothelial cells.

## 2. Common Mechanisms of *S. aureus* Binding Platelets and Endothelial Cells

The study of interaction between *S. aureus* and host cells is becoming an increasingly complex field largely because the bacterial interaction with host cells is not only species dependent but strain dependent also. This may be due to the wide repertoire of what are known as microbial surface components recognising adhesive matrix molecules (MSCRAMM’s) expressed on the bacterial surface [25]. It is through these MSCRAMM’s that *S. aureus* mediate either a direct or indirect interaction with host cells.

Direct interactions occur when *S. aureus* cell wall proteins (or equivalent recognition components) bind directly to an exposed host cell receptor. Typically these bacterial molecules display agonist like properties on the host receptor and binding usually, but not strictly, results in activation of the host cell. Examples of direct interactions occur when *S. aureus* protein A (SpA) binds directly to endothelial cell gC1qR/p33 receptor [26] or *S. aureus* iron-regulated surface determinant B (IsdB) binding platelet receptor GPII/bIIIa [27].

Indirect interactions require an accessory molecule, usually a plasma protein which is a natural ligand for a host receptor, to link *S. aureus* with the host cell. This is the case of fibrinogen crosslinking *S. aureus* fibronectin binding proteins (FnBPs) to endothelial cell α5β1[28] and *S. aureus* clumping factor A (ClfA) to platelet GPIIb/IIIa [15].

An alternative interaction occurs when *S. aureus* secretes a protein or toxin which binds directly to the host cell. Examples include; the secretion of extracellular adherence protein (EAP) which has the ability to bind to itself and also serves as a binding site for gC1qR/p33 [26] and; α-toxin, a pore forming toxin produced by *S. aureus* which has been shown to induce platelet activation leading to the assembly of the prothrombinase complex on the platelet surface [29].

## *3. Staphylococcus aureus* Interactions with Endothelial Cells

An early awareness of the bacterial response to the dynamic environment in which they find themselves upon entering the bloodstream, was established by Gould *et al.* Using punch biopsy, valve leaflets were excised from canine hearts and placed on a rotator to replicate the shear environment found in the heart. By radiolabelling the *S. aureus* with 51Cr, the authors could assess the extent of binding to the rotating heart valves using scintillation spectrometry [30]. Additionally, the authors homogenised the valve leaflets and quantified the number of bacteria adhered by colony forming units [31]. This was one of the first definitive sets of experiments that demonstrated bacterial binding to valvular endothelium as an important characteristic of bacteria colonisation in the bloodstream. Building on these initial observations Ogawa *et al.*, investigated the interaction between clinical isolates of *S. aureus* and human endothelial cells [32]. As with previous observations *S. aureus* was shown to be capable of binding to the endothelial cells. However, while early attempts were made to mimic the physiological dynamic conditions experienced in the vasculature, these experiments were carried out in the absence of whole blood. This is an important consideration to take into account, as the involvement of a plasma protein bridging the bacteria to the endothelial cells on the valve was not determined.

To address this, subsequent studies investigated the role of plasma proteins in *S. aureus* binding to human umbilical cord vascular endothelial cells (HUVECs). Using static binding assays, the authors demonstrated that *S. aureus* binding to HUVECs increased 4-fold in the presence of plasma. In an effort to identify the key plasma protein(s) involved in bridging *S. aureus* to HUVECs, endothelial cells were first coated with purified plasma proteins. Results from these studies clearly demonstrated that fibrinogen increased *S. aureus* binding 3-fold compared to when carried out in the absence of plasma. In contrast, there was only a slight increase in adherence to HUVECs when repeated in the presence of either fibronectin or vitronectin [33]. To further substantiate the role for fibrinogen the authors investigated adherence in the presence of afibrinogenemic plasma. Their studies indicated that *S. aureus* binding to HUVECs in the presence of afibrinogenemic plasma was no different to that of the control containing no plasma [34,35].

While significant steps were being made toward understanding the molecular mechanisms underlying *S. aureus* infection of the endothelium, considerable evidence to support the concept that immune and inflammatory mediators released at sites of infection, such as Tumour Necrosis Factor alpha (TNFα), Interleukin 1 beta (IL-1β) and interferon gamma (IFNγ), can act directly on endothelial cells to modulate function in pathologic processes. To this end, Cheung and colleagues determined that endothelial cells grown in the presence of TNFα for 4 hours, were 4 times more susceptible to *S. aureus* binding than in the absence of TNFα [36]. IL-1β and IFNγ showed no such effect. Consistent with previous findings these experiments were carried out in the presence of fibrinogen.

Infection in the bloodstream can also activate the coagulation cascade leading to the generation of thrombin, an additional molecule capable of modulating endothelial cell function [37]. Addition of α-thrombin to endothelial cells in the presence of fibrinogen, resulted in 2-4 fold increase in *S. aureus* binding. In an attempt to identify the region on fibrinogen responsible, the authors pre-incubated the endothelial cells with the inhibitory peptide Arg-Gly-Asp-Ser (RGDS). However this peptide failed to inhibit the interaction. Of key importance, the investigators sheared the endothelial cells at 600s-1, which is consistent with the physiological shear forces experienced in veins in the vasculature.

The success of *S. aureus* as a pathogen in the bloodstream is its ability to express a wide array of cell wall proteins on its surface [25]. Expression of these proteins depends on the growth phase of the bacteria. Tompkins *et al.*, demonstrated that *S. aureus* grown to the exponential phase adhered more efficiently to HUVECs than *S. aureus* grown to the stationary phase [38]. Trypsin treatment of *S. aureus* at the exponential phase abolished binding suggesting the proteins involved in binding to static HUVECs are trypsin sensitive. Using fast protein, peptide and polynucleotide liquid chromatography (FPLC) the authors identified proteins with molecular weights of 30, 55, 57, 70 and 85 kDa. Using a competition assay, the 55-57-kDA protein was identified as SpA. The authors suggested that SpA is not involved in the interaction as SpA-positive and -negative strains of *S. aureus* do not differ in their ability to bind to static HUVEC [38]. The 30, 70 and 85 kDa proteins were not identified in this study.

The virulence properties of *S. aureus* are controlled by global regulatory systems including Agr and Sar. Previous studies suggest that these loci primarily regulate the expression of cell wall proteins during the exponential phase of growth [39]. Using strains of *S. aureus* deficient in Agr, Sar and Agr/Sar expression, Shenkman and colleagues demonstrated a significantly reduced interaction with several plasma proteins and endothelial cells. Deletion of the *agr* locus significantly inhibited *S. aureus* adhesion to fibrinogen, thus preventing the bridging interaction with endothelial cells. Deletion of the *sar* locus, however, increased *S. aureus* adhesion to fibronectin, von Willebrand factor (vWF), extracellular matrix proteins and endothelial cells. Of particular interest is that the authors compared these effects under both static and shear conditions (200s-1) and concluded that under shear conditions the effects were far more pronounced [40]. In contrast to these studies Pohlmann-Dietze *et al.*, demonstrated that an isogenic *agr* mutant showed maximal adherence to static endothelial cells using a strain of *S. aureus* in the stationary phase of growth [41]. Capsular polysaccharide (CP) 5 and 8 are expressed on most *S. aureus* strains that colonise and infect humans and are important surface components regulated by Agr. The authors suggest that CP5 inhibits the adherence of *S. aureus* to static endothelial cells by masking a major cell wall adhesin that is expressed in the stationary phase of growth. Support for this theory lies with the demonstration that unencapsulated or Agr deficient strains adhere strongly to static endothelial cells.

Despite early studies suggesting that *S. aureus* major cell wall determinant SpA was not involved in adherence to endothelial cells, subsequent results challenge these findings. SpA binds to a variety of ligands including plasma proteins IgG [42] and vWF [43] as well as a number of host receptors, including tumor necrosis factor receptor-1 [44], epidermal growth factor receptor [45] and complement receptor gC1qR/p33 [46]. Edwards *et al.*, demonstrated that blocking gC1qR/p33 on statically grown endothelial cells using a monoclonal antibody significantly reduced binding [26]. As SpA is a ligand for gC1qR/p33, a strain deficient in its expression which significantly reduced binding to the endothelial cells was used. Interestingly, gC1qR/p33 is only expressed on the surface of endothelial cells upon activation [47]. It is currently unclear if immune or inflammatory mediators are the only signals that activate endothelial cells. It is conceivable however, that initial communication between *S. aureus* and endothelial cells can result in an outside-in signal leading to endothelial cell activation. Consistent with early observations for the necessity of focusing on the immunomodulatory role of cytokines at the site of infection, Edwards added TNFα for 4 hours pre-exposure to *S. aureus*. The resulting upregulation of gC1qR/p33 on the surface of the static endothelial cells facilitated binding [26]. Upon endothelial cell activation the Weibel Palade Bodies are transported to the surface of the cell thus releasing vWF making it available for interaction with *S. aureus*. SpA has previously been shown to bind to vWF [43] although its role, if any, in bloodstream infection is not yet clear. *S. aureus* expresses a von Willebrand factor binding protein (vWfbp). Using a parallel flow chamber, Claes *et al.*, demonstrated that adhesion under high shear conditions (1000s-1) was significantly reduced using a strain of *S. aureus* deficient in expression of vWfbp or by blocking the A1 domain of vWF. Using a venous mesenteric perfusion mouse model the authors also demonstrated that calcium ionophore A23187 induced activation of endothelial cells leading to vWF exposure on the cell surface. This caused a significant deposition of *S. aureus* on the blood vessel. Deletion of vWfbp reduced this interaction [48].

EAP is a protein that binds several host glycoproteins and has both pro- and anti-inflammatory activity. Although EAP is secreted from *S. aureus* it can bind onto the surface of the bacteria and serve as a binding site for intercellular adhesion molecule-1 (ICAM-1), a protein expressed on the surface of endothelial cells [49]. EAP is also suggested to be involved in the internalisation of *S. aureus* into the endothelial cells [50], however others dispute this, claiming the absence of EAP confers to higher internalisation [51]. *S. aureus* internalisation into host cells bears significant resemblance to complement-mediated phagocytosis by professional phagocytes. While it is still unclear why *S. aureus* invade endothelial cells it is most likely for survival reasons, as neither immune cells or antibiotics can reach them once they have internalised. *S. aureus* invasion into endothelial cells is a highly controlled event involving F-actin rearrangement [52], tyrosine phosphorylation [53], mitogen activated protein kinase activation [54] and *src* family kinase activation [28,55]. Both formaldehyde fixed and live bacteria are equally invasive suggesting that no active bacterial process is involved in the internalisation. Results suggest that fibronectin is critical for invasion, as its removal or the use of a *S. aureus* strain defective of FnBP expression significantly reduces internalisation [52]. Endothelial cells express two known receptors for fibronectin, namely α5β1 and αVβ3. Inhibition of αVβ3 failed to have any effect on internalisation. However, using a monoclonal antibody against α5β1 significantly reduced internalisation of *S. aureus* into endothelial cells [56].

It is clear that as a consequence of *S. aureus* binding to endothelial cells a number of dysfunctional responses are initiated. For example, *S. aureus* has been shown to induce apoptosis in endothelial cells after 1 hour of infection. Esen *et al.*, demonstrated that *S. aureus* mediated endothelial cell apoptosis involved activation of caspases, acid sphingomyelinase and the Jun NH2-terminal kinase [57]. Though the trigger for apoptosis is still unclear, there is some suggestion that apoptosis is triggered following internalisation and others have demonstrated that α-toxin might be responsible [58]. Interestingly, while formaldehyde fixation of *S. aureus* fails to affect internalisation, it abolishes their ability to induce apoptosis, suggesting that an active microbial process involving viable bacteria is required to induce cell death. *S. aureus* binding to endothelial cells also triggers a strong inflammatory response. Several reports have demonstrated that following *S. aureus* infection, endothelial cells become more adhesive to leukocytes and monocytes as a result of an up-regulation in various cell adhesion molecules [59]. The release of a large number of chemokines leads to the recruitment of these cells to the site of infection [60]. This supports the concept that upon binding to endothelial cells, *S. aureus* induces a sustained inflammatory response consistent with the clinical signs of bloodstream infection. Vascular permeability is a further problem following *S. aureus* infection. Indeed one of the cardinal signs of sepsis is progressive subcutaneous and body cavity oedema typically caused by permeabilisation of the vascular endothelial cell monolayer [18]. Recent work from Powers and colleagues showed that vascular injury also occurs with interaction of α-toxin with A Disintegrin and Metalloprotease 10 (ADAM10) and subsequent cleavage of vascular endothelial cadherin, a process that is independent of *S. aureus* binding the endothelium [61,62]. To date, a common feature among the functional dysregulation of endothelial cells following *S. aureus* infection is that the molecular mechanisms resulting in these processes have not yet been fully elucidated. In addition, most studies have been carried out on HUVEC cells. The choice for a readily accessible source of endothelial cells has prevailed over the need to reproduce the heterogeneity of the endothelium and hence the potential preference of *S. aureus* for specific vascular beds. This, undoubtedly, contributes to the limitations in elucidating *S. aureus*-endothelial cells interactions [63,64].

## *4. Staphylococcus aureus* Interactions with Platelets

Most of the early reports into *S. aureus* interacting with platelets demonstrate that products released from bacteria were responsible for triggering platelet activation. In 1964 Siegel and Cohen made two critical observations; first, addition of α-toxin isolated from *S. aureus* led to the loss of single platelets as evidenced by turbidimetric aggregometry. Secondly, addition of α-toxin to human platelets resulted in leakage of intracellular ions; NAD+, K+ and ATP, though interestingly this was not the case with proteins, suggesting that α-toxin was not lysing the platelets [65]. Additional studies by Arvand and colleagues demonstrated that α-toxin induced secretion of platelet granules and most importantly procoagulant mediators, platelet factor 4 and factor V. Secreted factor V in turn associates with the platelet membrane leading to assembly of the prothrombinase complex [29]. This explains the major pathway responsible for the procoagulatory effects of α-toxin. *S. aureus* can release a component of its cell wall called lipoteichoic acid (LTA). Work by Sheu *et al.*, and later Waller *et al.*, demonstrated that LTA from *S. aureus* inhibited human platelet aggregation, calcium mobilisation and cyclic AMP [66,67], the latter group identifying the platelet activating factor receptor (PafR) as the inhibitory receptor on platelets [68]. Additional effects on platelet function induced by α-toxin have been recently revealed by Powers and colleagues. The mechanism involves ADAM10-mediatated glycoprotein VI proteolysis, which causes a reduction in platelet binding to collagen and fibrinogen. As a result, endothelial repair by platelets is impaired [69].

There are numerous cell wall proteins expressed on the surface of *S. aureus* that have been demonstrated to bind to and activate platelets. The majority of these cell wall proteins bind plasma proteins and bridge to a platelet receptor. SpA is found on greater than 90% of *S. aureus* strains. Initial reports demonstrated that SpA is capable of binding to immunoglobulin G (IgG) which bridges to the platelet antibody receptor, FcγRIIa [70]. This interaction results in platelet signal generation, GPII/bIIIa dependent platelet aggregation and serotonin release from the platelet dense granules. SpA can also bind to the A1 domain of vWF [43] which serves as a receptor for GPIbα expressed on platelets [71].

In addition to SpA, ClfA was demonstrated to promote attachment of *S. aureus* to platelets [72]. Furthermore O’Brien and colleagues showed that multiple cell wall proteins expressed on *S. aureus* have the ability to interact with and trigger platelet aggregation [73]. *S. aureus* cell wall protein ClfA and clumping factor B (ClfB) are shown to bind to fibrinogen, IgG and complement, which in turn bridge the bacterial protein to specific platelet receptors GPII/bIIIa, FcγRIIa and gC1qR/p33 resulting in receptor clustering and platelet activation [74]. FnBP plays a key role in inducing platelet aggregation. The mechanism through which Fibronectin binding protein A (FnbpA) induces platelet aggregation is more or less identical to that of the ClfA protein. FnbpA binds fibrinogen and IgG, bridging *S. aureus* to GPIIb/IIIa and FcγRIIa to trigger platelet activation and aggregation [75]. Finally *S. aureus* expresses a highly glycosylated serine rich protein called SraP on its surface [76]. Strains deficient in SraP expression are shown to have reduced virulence in a rabbit model of endocarditis. Currently, neither the plasma protein bridging molecules nor the platelet receptor to which SraP binds has been identified.

A growing concern about studies to date is the apparent lack of contrast with conditions experienced physiologically. For example, most of the above experiments have been carried out under static conditions or are not truly representative of the fluid shear environment experienced in the vasculature. To address this, several attempts have been made to reproduce rheological parameters to create an environment that mimics the conditions experienced in the circulation, resulting in sheared conditions at physiological rates. Pawar *et al.*, demonstrated that when *S. aureus* is mixed with whole blood isolated from healthy individuals, thrombus formation occurred. Similar to the results described above, thrombus formation was dependent on multiple *S. aureus* cell wall proteins including SpA, ClfA, SdrC, SdrD and SdrE [77]. A limitation to using a cone and plate viscometer is that it measures thrombus formation in a soluble setting. However thrombus formation on a heart valve in IE occurs under stable conditions. To address this *Kerrigan et al.*, developed a parallel flow chamber to assess the interaction between *S. aureus* and platelets in whole blood. Using this method, the authors demonstrated that platelets perfused over immobilised *S. aureus* under arterial shear led to a very strong adhesion, followed by rapid aggregate formation. Deletion of ClfA (though not SpA or FnbpA) from *S. aureus* abolished adhesion and subsequent aggregate formation. Thrombus formation was dependent upon combined addition of fibrinogen and specific immunoglobulin to the plasma-free system which led to platelet adhesion followed by aggregate formation. This highlights the importance of these plasma proteins in this process. Interestingly platelets did not adhere to or induce aggregate formation under low shear (venous) conditions using the parallel flow chamber [15].

In an attempt to further correlate better with *in vivo* conditions, recent attention has focused on the process of growing bacteria in culture in the laboratory. The blood stream is an environment in which iron is sequestered in haem- or haemoglobin. Recent studies have demonstrated that the lack of iron available *in vivo* inactivates the Fur repressor in *S. aureus* causing an up-regulation of a number of genes that typically wouldn’t be expressed when growing in normal laboratory bacterial growth media. This has led to the identification of a growing family of iron-regulated surface determinant proteins that have been recently identified in *S. aureus* grown in iron limited conditions. Miajlovic *et al.*, demonstrated that IsdB can bind directly (in the absence of plasma proteins) to the purified platelet fibrinogen receptor GPIIb/IIIa to support platelet adhesion and induce platelet aggregation. A *S. aureus* strain defective in IsdB expression displayed a reduced ability to adhere to or induce platelet aggregation [27]. Collectively these more recent studies highlight the importance of focusing on more physiological methods for studying platelet bacterial interactions.

## 5. Conclusions

Significant progress has been made in our understanding of molecular mechanisms leading to bloodstream infection. However, much work is still required as research has of yet failed to identify a novel treatment. The current management plan for bloodstream infection focuses on reducing the infection through the use of aggressive intravenous antibiotic therapy often delivered in high concentrations for long periods of time. Antibiotics are not a reliable long term solution due to the rapid global emergence of antibiotic resistant strains of bacteria. Indeed, according to the most recent report on antimicrobial resistance by the World Health Organisation a “post-antibiotic” era is imminent, suggesting that the use of antibiotics in bloodstream infection will shortly come to an abrupt end. It is therefore pertinent that greater efforts are placed on determining the molecular mechanisms through which *S. aureus* triggers dysfunction in both endothelial cells and platelets representing a more physiological environment such as that experienced under shear conditions. A more complete understanding in these mechanisms will lead to the development of novel therapies to treat this infection.
